# Thermodynamics Analysis of Refinery Sludge Gasification in Adiabatic Updraft Gasifier

**DOI:** 10.1155/2014/758137

**Published:** 2014-02-03

**Authors:** Reem Ahmed, Chandra M. Sinnathambi, Usama Eldmerdash, Duvvuri Subbarao

**Affiliations:** ^1^Department of Chemical Engineering, Universiti Teknologi PETRONAS, 31750 Tronoh, Perak, Malaysia; ^2^Fundamental and Applied Sciences Department, Universiti Teknologi PETRONAS, Bandar Seri Iskandar, 31750 Tronoh, Perak, Malaysia

## Abstract

Limited information is available about the thermodynamic evaluation for biomass gasification process using updraft gasifier. Therefore, to minimize errors, the gasification of dry refinery sludge (DRS) is carried out in adiabatic system at atmospheric pressure under ambient air conditions. The objectives of this paper are to investigate the physical and chemical energy and exergy of product gas at different equivalent ratios (ER). It will also be used to determine whether the cold gas, exergy, and energy efficiencies of gases may be maximized by using secondary air injected to gasification zone under various ratios (0, 0.5, 1, and 1.5) at optimum ER of 0.195. From the results obtained, it is indicated that the chemical energy and exergy of producer gas are magnified by 5 and 10 times higher than their corresponding physical values, respectively. The cold gas, energy, and exergy efficiencies of DRS gasification are in the ranges of 22.9–55.5%, 43.7–72.4%, and 42.5–50.4%, respectively. Initially, all 3 efficiencies increase until they reach a maximum at the optimum ER of 0.195; thereafter, they decline with further increase in ER values. The injection of secondary air to gasification zone is also found to increase the cold gas, energy, and exergy efficiencies. A ratio of secondary air to primary air of 0.5 is found to be the optimum ratio for all 3 efficiencies to reach the maximum values.

## 1. Introduction

Petroleum refineries produce a lot of oily sludge and are classified as “Hazardous Waste” under Schedule Waste 2. This sludge usually contains heavy oxyhydrocarbons, traces of heavy metals such as Cd, Cu, Zn, Mn, Ni, and Pb, and water. Several conventional technologies, such as landfill and biological treatment, have been implemented to handle the waste [1]. This can lead to groundwater contamination as well as air pollution due to volatile organic chemicals, odor problem, fire hazard, and adverse health effects [2]. Combustion and incineration can help recover some of the energy and generate greenhouse gases. Instead, gasification by partial oxidation of the oily sludge can convert the hazardous material into syngas rich in H_2_ and CO while generating some energy as well. The gasification of a fuel involves converting the chemical energy contained in the fuel into chemical products as well as sensible energy of the produced gas. According to the first law of thermodynamics, energy can never be lost. However, according to the second law of thermodynamics energy conversion processes are accompanied by an irreversible increase in entropy, which leads to a decrease in exergy (available energy). Thus, even though the energy is conserved, the quality of energy decreases because energy is converted into a different form of energy, from which less work can be obtained [3].

Zhang et al. [4] have evaluated the biomass gasification with air in autothermal gasifiers from energy and exergy aspect. They have used two factors (ER and gasification temperature) to study the energy and exergy distribution and efficiencies. Their results indicate that the chemical energy values of product gases from biomass are 2.16–5.20 times higher as the corresponding physical energy values, while the chemical exergy values are 4.50–13.45 times as the corresponding physical exergy values. The energy and exergy efficiencies of biomass gasification are, respectively, in ranges of 52.38–77.41% and 36.5–50.19% and mainly increase and then decline when ER or gasification temperature increases.

Mhilu [5] has derived a thermodynamic equilibrium model to predict the main product gas composition CO, CO_2_, H_2_, and CH_4_ for gasification of different biomass materials. The gasification regime is investigated at temperatures ranging from 800 K to 1400 K and at equivalence ratio (ER) values between 0.3 and 0.4. Results indicate that the application of preheated air has an effect on the increase of the chemical exergy efficiency of the product gas, hence reducing the level of irreversibility. Similarly, these results show that the combined efficiency based on physical and chemical exergy is low, suggesting that higher irreversibilitys are encountered, since the exergy present in the form of physical exergy is utilized to heat the reactants. Such exergy losses can be minimized by altering the ratio of physical and chemical exergy in the syngas production.

Karamarkovic et al. [6] have found that the preheating of gasifying air by heat exchanging with the product gas is beneficial for the energetic and exergetic efficiencies of the gasification process. The higher the preheating temperature the larger their efficiencies.

Reddy [7] has discussed the gasification of coal and biomass for power generation systems. The exergy was analyzed to identify the irreversibility and the ways to improve the performance of power generation systems. The exergy analysis is becoming very important to improve the performance and design of energy system components and overall systems. Any improvement in the energy systems based on second law will result in reduction in greenhouse gas emissions leading to reduced global warming.

The objectives of this paper are therefore to quantify the cold gas, energy, and exergy efficiencies of dry refinery sludge gasification in updraft reactor at different ER. It is also to find out whether these efficiencies could be maximized by using secondary air injected to gasification zone at various ratios. The chemical and physical energy and exergy of the producer gas are also investigated and compared with other biomass fuels.

## 2. Adiabatic Updraft Gasifier

**Figure 1:**
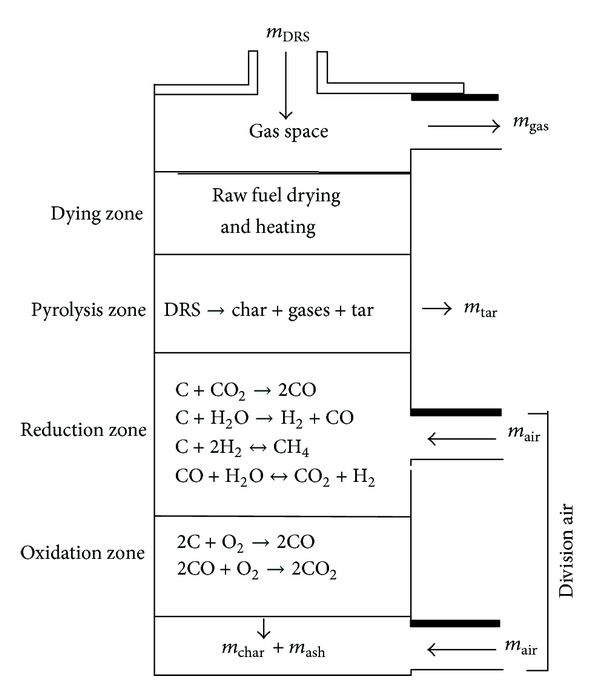
Schematic diagram for exothermic and endothermic reactions in adiabatic updraft gasifier.

The updraft gasifier unit used in this study was made from mild steel and cement with 25 mm thickness and volume of 0.16 m^3^. The setup of reactor rig is described by Konda et al. [8].  Figure 1 shows the exothermic and endothermic reactions in adiabatic updraft gasifier. In the drying process, the moisture in the solid fuel evaporates. The pyrolysis process separates the water vapor, organic liquids, and noncondensable gases from the char or solid carbon of the fuel. The combustion process oxidizes fuel constituents in an exothermic reaction, while the gasification process reduces them to combustible gases in an endothermic reaction. The four major gasification reactions are water-gas reaction, Boudouard reaction, shift conversion, and methanation. These are the most important reactions which could produce the syngas.

## 3. Thermodynamic Analysis

### 3.1. Mass Conversation

Mass balance or material balance is essential to validate the experimental results of the gasifier. However, to perform a mass balance for an updraft gasifier, input and output materials should be visualized. The input streams to the gasifier include DRS, dry air, and their moistures, whereas the output streams include producer gas, char, and tar.

Applying the law of conservation of mass to the gasification process yields [9]:

(1) $$\begin{array}{c} \Sigma M_{i}=\Sigma M_{o}. \end{array}$$

Here the overall mass balance and its closure are calculated for DRS gasification in updraft reactor at different ER. The mass entering the updraft gasifier contains DRS and air. The products of gasification are product gas, tar, ash, and char or unreacted carbon. An overall efficiency (in terms of cold gas efficiency) of the gasification system was obtained through mass balance which could be performed for the input and output streams of the gasification process as shown in the equations below.

The mass conservation embodies

(2) $$\begin{array}{c} m_{\text{DRS}}+m_{\text{air}}=m_{\text{gas}}+m_{\text{tar}}+m_{\text{char}}+m_{\text{ash}}, \end{array}$$

where *m*_DRS_, *m*_air_, *m*_gas_, *m*_tar_, *m*_char_, and *m*_ash_ denote the mass rates of DRS, air, product gas, tar, char, and ash, respectively.

### 3.2. Heat Conversation

According to energy conservation law, the corresponding energy balance of an updraft gasifier can be written as

(3) $$\begin{array}{c} \text{E}{\text{n}}_{\text{DRS}}+\text{E}{\text{n}}_{\text{Air}}⟶\text{E}{\text{n}}_{\text{Gas}}+\text{E}{\text{n}}_{\text{char}}+\text{E}{\text{n}}_{\text{Tar}}+\text{E}{\text{n}}_{\text{Loss}}, \end{array}$$

where En_DRS_, En_Air_, En_Gas_, En_char_, En_Tar_, and En_Loss_ represent the energy rates of DRS, air, product gas, char, tar, and the lost part, respectively. En_Loss_ relates to the energy from ash and the lost heat [4].

The total energy of a stream flow is

(4) $$\begin{array}{c} {\text{En}}^{\text{to}}={\text{En}}^{\text{ki}}+{\text{En}}^{\text{po}}+{\text{En}}^{\text{ph}}+{\text{En}}^{\text{ch}}. \end{array}$$

Here, En^to^, En^ki^, En^Po^, En^ph^, and En^ch^ represent the total, kinetic, potential, physical, and chemical energy rates of the stream, respectively. Neglecting

(5) $$\begin{array}{c} {\text{En}}^{\text{ki}}=\left(\frac{{mv}^{2}}{2}\right),\\ {\text{En}}^{\text{Po}}=mgz. \end{array}$$

Equation (4) reduces to

(6) $$\begin{array}{c} {\text{En}}^{\text{to}}={\text{En}}^{\text{ph}}+{\text{En}}^{\text{ch}}. \end{array}$$

For combustible gases,

(7) $$\begin{array}{c} \text{E}{\text{n}}^{\text{ph}}=m_{\text{gas}}h, \end{array}$$

(8) $$\begin{array}{c} \text{E}{\text{n}}^{\text{ch}}=m_{\text{gas}}\text{HHV}, \end{array}$$

(9) $$\begin{array}{c} \text{E}{\text{n}}^{\text{to}}=m_{\text{gas}}\left(h+\text{HHV}\right), \end{array}$$

where *m*_gas_, *h*, and HHV represent the mass flow rate, specific enthalpy, and high heating value of the gas, respectively. The above equations (7) and (8) are suitable for air, product gas, and tar [4]. The mass flow rate of gases is calculated from mass balance and producer gas yield.

The yield of gas in Nm^3^ kg^−1^ [10]:

(10) $$\begin{array}{c} {\text{Y}}_{\text{gas}}=\frac{m_{a}\times 0.79}{m_{D}\left(1-X_{\text{ash}}\right)\times {\text{N}}_{2}\%}, \end{array}$$

where *m*_*a*_, *m*_*D*_, *X*_ash_, and N_2_% are flow rate of air m^3^ hr^−1^, mass flow rate of DRS kg hr^−1^, mass fraction of ash, and volume percentages of N_2_.

For air,

(11) $$\begin{array}{c} \text{E}{\text{n}}^{\text{to}}=m_{\text{air}}h. \end{array}$$

While for DRS and char (unreacted carbon), the total energy can be simplified to

(12) $$\begin{array}{c} \text{En}=m_{D}\text{HHV}. \end{array}$$

The specific enthalpy of a component is

(13) $$\begin{array}{c} h=h_{0}+\int_{T_{0}}^{T}CpdT, \end{array}$$

where *h* and *h*_0_ represent the specific enthalpy at the designated temperature (*T*) and the environmental temperature (*T*_0_), respectively. The specific enthalpy values of some gases at the environmental temperature are shown in Table 1. *C*_*p*_ is the constant pressure specific heat capacity in kJ kmol^−1^ K^−1^. 

The empirical equation is

(14) $$\begin{array}{c} C_{p}=a+bT+{cT}^{2}+{dT}^{3}. \end{array}$$

The coefficients *a–d* of constant pressure specific heat capacity of some gases are given in Table 1.

The high heating value of DRS (HHV biomass) was experimentally measured, whereas the LHV of DRS is obtained by the following 6 correlation in MJ kg^−1^ [4]:

(15) $$\begin{array}{c} \text{HHV}=\text{LHV}+21.978\text{H}. \end{array}$$

Here, *H* is the weight fraction of element *H* in the ultimate analysis. The heating value of the synthesis gas was evaluated in terms of higher heating value, HHV, and lower heating value, LHV, at standard temperature and pressure and can be determined by considering the volumetric percentage of the gas constitutes (CO, H_2_, and CH_4_) and can be estimated from the following, respectively [10, 11]:

(16) HHV=[H2%×30.52+CO%×30.18+CH4%×95]×4.1868LHV=[H2%×25.7+CO%×30+CH4%×85.5]×4.2.

### 3.3. Exergy Analysis

The exergy balance can be written as

(17) $$\begin{array}{c} \text{E}{\text{x}}_{\text{DRS}}+\text{E}{\text{x}}_{\text{Air}}=\text{E}{\text{x}}_{\text{Gas}}+\text{E}{\text{x}}_{\text{Tar}}+\text{E}{\text{x}}_{\text{char}}+\text{E}{\text{x}}_{\text{Loss}}, \end{array}$$

where Ex_DRS_, Ex_Air_, Ex_Gas_, Ex_Tar_, and Ex_char_ represent the exergy rates of DRS, air, product gas, tar, and char, respectively. Ex_Loss_ denotes the exergy rate lost from this system, and it includes the exergy from ash, lost heat, and irreversibility of the process.

The exergy of the product gas is comprised of two components: exergy physical Ex^ph^ and chemical exergy Ex^ch^. Neglecting the kinetic and potential exergy,

(18) $$\begin{array}{c} {\text{Ex}}^{\text{to}}={\text{Ex}}^{\text{ph}}+{\text{Ex}}^{\text{ch}}. \end{array}$$

Here, Ex^ph^ and Ex^ch^ represent the physical and chemical exergy rates of the stream, respectively. The physical exergy is the maximum theoretical work obtainable as the system passes from its initial state where the temperature is the gasifying temperature and the pressure equals the gasifier pressure to the restricted dead state where the temperature is *T*_0_ and the pressure is *P*_0_ [12].

The physical exergy of a pure compound of a mixture can be easily calculated using enthalpy and entropy data for the given system and is given by the expression

(19) $$\begin{array}{c} {\text{Ex}}^{\text{ph}}=m\left(h-h_{0}\right)-T_{0}\left(S-S_{0}\right), \end{array}$$

where *m* is mass flow rate of the stream in K mol s^−1^ and *S* and *S*_0_ denote the specific entropy in kJ kmol^−1^ K^−1^ at the specified state (*P* and *T*) and the environmental condition (*P*_0_ = 1 atm and *T*_0_ = 298 K), respectively. The specific entropy values of some gases are in Table 1.

(*S* − *S*_0_) or Δ*s* is calculated from the equation below:

(20) $$\begin{array}{c} \Delta S=Cp\cdot \ln\left(\frac{T_{2}}{T_{1}}\right). \end{array}$$

The chemical exergy is the maximum theoretical useful work obtainable as the system passes from the restricted dead state to the dead state where it is in complete equilibrium with the environment [12]. The chemical exergy of the mixture *ε*_0,*m*_ is determined by the composition and concentration of components in the mixture and is given by

(21) $$\begin{array}{c} \varepsilon_{0,m}=\sum_{i}x_{i}\varepsilon_{0,i}+RT_{0}\sum_{i}x_{i}\ln x_{i}, \end{array}$$

where *ε*_0,*i*_ is the standard chemical exergy of the material in kJ kmol^−1^. The standard chemical exergy of a pure chemical compound is equal to the maximum amount of work obtainable when a compound is brought from the environmental state, characterized by the environmental temperature *T*_0_ and environmental pressure *P*_0_, to the dead state, characterized not only by the same environmental conditions of temperature and pressure, but also by the concentration of reference substances in standard environment [3]. The standard chemical exergy values of some components are given in Table 1.

It should be noticed that the chemical exergy of the mixture is always lower than the sum of exergy of individual components, as the second term in the above equation is always negative.

For air the chemical exergy rate is defined as

(22) $$\begin{array}{c} {\text{Ex}}^{\text{ch}}=m\cdot \varepsilon_{0,i}, \end{array}$$

where *m* is stream flow rate in Kmol s^−1^ and *ε*_0,*i*_ (kJ Kmol1^−1^) is the standard chemical exergy of the component given in Table 1.

For the DRS fuel, thermodynamic properties are not available. Therefore, the statistical correlation of Szargut and Styrylska [13] was used:

(23) $$\begin{array}{c} {\text{Ex}}_{\text{DRS}}=\beta \cdot {\text{LHV}}_{\text{DRS}}\cdot {\text{y}}_{\text{DRS}}. \end{array}$$

Here, *β* is a correlation factor and can be calculated from Szargut [14]:

(24) β=((1.044+0.016HC−0.3493OC  ×(1+0.0531HC)+0.0493NC))×((1−0.4124OC))−1.

Ex_DRS_ is the chemical exergy of DRS. LHV and *Y*_DRS_ are the lower heating value (MJ kg^−1^) and ash free fraction of DRS, respectively. C, H, O, and N are the weight fractions of carbon, hydrogen, oxygen, and nitrogen in the ultimate analysis of biomass, respectively.

The DRS considered has a higher heating value of 26.6 kJ g^−1^ and lower heating value calculated from (15) is 24.99 kJ g^−1^. Consider

(25) $$\begin{array}{c} y_{\text{DRS}}=1-y_{\text{ash}}, \end{array}$$

*y*_ash_ is the fraction of ash in the feedstock which was found to be 12.1% from the proximate analysis.

### 3.4. Energy and Exergy Efficiencies

To comprehensively evaluate biomass gasification, both energy and exergy efficiencies are introduced. They are defined as

(26) $$\begin{array}{c} \eta_{\text{En}}\left(\frac{{\text{En}}_{\text{gas}}}{\left({\text{En}}_{\text{DRS}}+{\text{En}}_{\text{air}}\right)}\right)\times 100\%\\ \eta_{\text{Ex}}\left(\frac{{\text{Ex}}_{\text{gas}}}{\left({\text{Ex}}_{\text{DRS}}+{\text{Ex}}_{\text{air}}\right)}\right)\times 100\%, \end{array}$$

where *η*_En_ is the energy efficiency (gas energy divided by total energy input) and *η*_Ex_ is the gas exergy divided by the total input exergy.

### 3.5. Gasification Efficiency

Gasification efficiency is one of the important factors that determine the actual technical operation. It usually depends on the gasifier type and design as well as on the characteristics of the fuel. The gasification efficiency in this study was expressed in terms of cold gas efficiency [8, 9]:

(27) $$\begin{array}{c} \text{CGE}=\left[\frac{Y_{\text{gas}}\left(\text{N}{\text{m}}^{3}\;\text{k}{\text{g}}^{-1}\right)\times \text{HH}{\text{V}}_{\text{gas}}\left(\text{MJ}\;\text{N}{\text{m}}^{-1}\right)}{\text{HH}{\text{V}}_{\text{fuel}}\left(\text{MJ}\;\text{k}{\text{g}}^{-1}\right)}\right]\times 100, \end{array}$$

where *Y*_gas_ is the fuel gas production, HHV_gas_ is the higher heating value of the producer gas, and HHV_fuel_ is the higher heating value of the DRS.

### 3.6. Equivalent Ratio

The equivalent ratio reflects the combined effect of the air flow rate, flow rate of DRS fuel and duration of the test. The equivalence ratio for this study was calculated by:

(28) $$\begin{array}{c} \text{ER}=\frac{m_{a}\times t}{M_{\text{DRS}}\times \text{A}{\text{F}}_{\text{stoich}}}, \end{array}$$

where  *m*_*a*_ is the air flow rate (m^3^/h), *t* is the duration of the experiment (*h*), *M*_DRS_ is mass input of DRS fuel (kg), and AF_stoich_ is the air fuel ratio at stoichiometric conditions (7.75 m^3^ of air per kg of DRS).

## 4. Results and Discussions

### 4.1. Mass and Energy Balance and Closure

The overall mass and energy balance at different equivalence ratios and their closure are computed and presented in Tables 2 and 3, respectively. From Table 2 the average mass closure is found to be 0.96, while the average energy closure (Table 3) is 0.59. Ideally, mass and closure are expected to be unity since input should be equal to output for both. However, mass closure was found to be more than 1 for some experiments which might be due to some instrumental errors as well as the residual biomass inside the gasifier, which could not be measured due to operational difficulties.

Further, the size of the gasifier contributed to these discrepancies in mass closures because of the higher probability that significant amount of refinery sludge may be retained in the gasifier after the completion of experiment. The tar production during gasification of DRS is very sticky and it is not easy to measure the total mass of tar and this introduces error in mass measurements.

Mass closure increases with ER while further increase in ER or air flow rate causes decrease in mass closure; this might be due to reduction of producer gas as the air flow increases. Further increase in ER resulted in increase in the oxidation process over the reduction process and this may reduce the amount of char production. From Table 3 a similar behavior was observed; the energy closure increases as ER increases until it reached the maximum closure of 0.75 at ER 0.195 then decreases dramatically. The lower value of energy closure might come from instrument design or construction error resulting in losing some of energy. Furthermore, the higher the ER value, the higher the N_2_ (inert gas) percentages diluting the combustible gases which may lead to reducing the energy of the producer gas.

### 4.2. Effect of ER on Cold Gas, Energy, and Exergy Efficiencies

**Figure 2:**
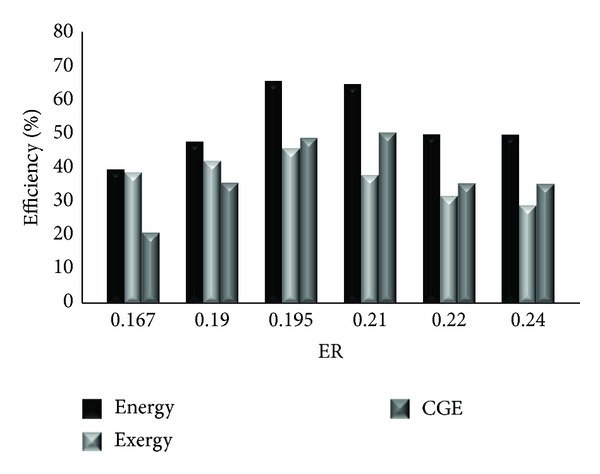
Influence of ER of on cold gas, energy and exergy efficiencies.

The influence of ER on cold gas, energy, and exergy efficiencies of product gases for dry refinery sludge gasification in adiabatic updraft gasifier is shown in Figure 2.

The maximum energy and exergy efficiencies of DRS gasification are between 72.44% and 50.38%, respectively, at ER of 0.195. Further increase in ER caused a sharp decrease in energy and exergy efficiencies. This was due to the increase of the equivalence ratio as a result of more O_2_ being supplied to the gasifier, which increased the gasification temperature, hence accelerating the gasification process and improving the gas quality. Further increase in the equivalence ratio provided more N_2_ with air and diluted the producer gas, which degraded the gas quality. It is also observed that the cold gas efficiency (CGE) had the same trend as that of energy and exergy; with an increase in the equivalence ratio from 0.167 to 0.21, the CGE increased from 22.97 to 55.47% and then decreased to 38.88% at maximum equivalence ratio (ER 0.24). The CGE depended upon the gas yield and the volumetric percentage of CO, CO_2_, and CH_4_ in the producer gas.

It is found that the cold gas and energy efficiencies are much higher than the corresponding exergy efficiencies, which is in agreement with [4]. They have found that the energy efficiencies of biomass gasification are between 52.38% (rice husk, ER = 0.25) and 77.41% (wood chip, ER = 0.38), while those of polypropylene gasification are from 54.45% (ER = 0.20) to 58.43% (ER = 0.35). The exergy efficiencies of biomass gasification are between 36.5% (rice husk, ER = 0.25) and 50.19% (wood chip, ER = 0.38). The exergy efficiencies of dry refinery sludge gasification are from 31.93% (ER = 0.24) to 50.38% (ER = 0.195). In Figure 2 when ER increases from 0.167 to 0.195, the cold gas, energy, and exergy efficiencies rise monotonously. We can foresee that these efficiencies will be reduced by the increasing N_2_ which has low energy, and exergy values [4]. Resulting from the dilution of N_2_, both the energy and exergy efficiencies will definitely decline when ER is high enough.

Generally speaking, the typical trend is that the cold gas, energy, and exergy efficiencies increase first and then reduce when ER increases. Hence, a proper ER should be employed to get higher efficiencies for all of them. In this work, the optimum ER for dry refinery sludge gasification in updraft reactor seems to be in 0.195.

### 4.3. Producer Gas Energy and Exergy Distributions

**Figure 3:**
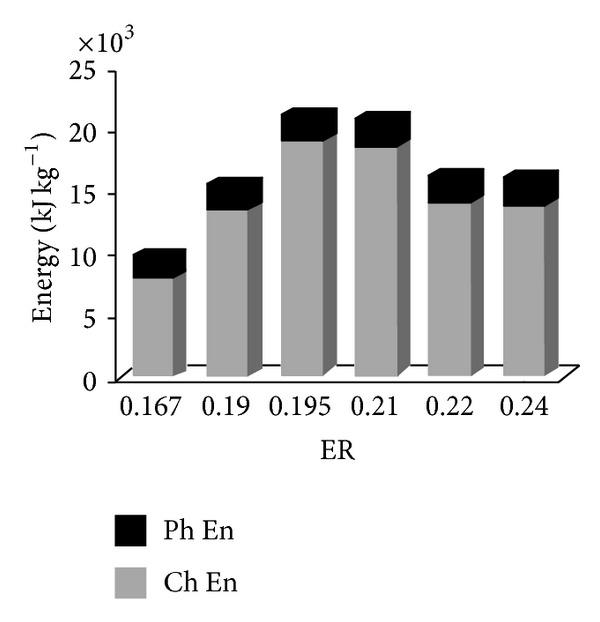
Effect of ER on energy distribution of product gas.

Figure 3 exhibits the energy distribution of product gases for dry refinery sludge gasification. It can be observed that the chemical energy values of product gases are much higher than the corresponding physical energy values. From the same figure it is found that the chemical energy values are 10.131 (ER = 0.195) times as the corresponding physical energy values. This relationship is mainly resulted from the fact that product gases have much higher heating values than the corresponding enthalpy values.

This result is in agreement with Zhang et al. [4], where they found that the chemical energy values are 2.16 (rice husk, ER = 0.35)–5.20 (wood chip, ER = 0.32) times as the corresponding physical energy values for biomass, while 2.91 (ER = 0.45)–8.26 (ER = 0.20) for polypropylene. This relationship is mainly resulted from the fact that product gases have much higher heating values than the corresponding enthalpy values.

Based on 1 kg of DRS, the total energy values of product gases are between 20,856–9,474 KJ being much higher than the other biomass fuel. This is because DRS has much higher carbon and hydrogen in the ultimate analysis than those of biomass. The carbon and hydrogen weight percentages (dry ash-free basis) in DRS are, respectively, 56.72% and 7.8%, while those in biomass, respectively, range from 46.4–50.0% to 5.7–6.775%, [4]. Hence, DRS has a higher heating value than biomass 26.6 MJ/kg.

**Figure 4:**
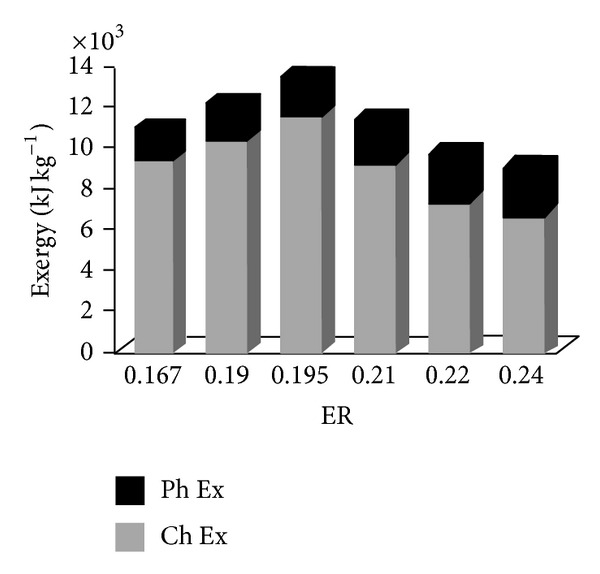
Effect of ER on exergy distribution of product gas.

In Figure 4, the chemical exergy values of product gases are 5.709 (ER = 0.195) times than the corresponding physical exergy values for biomass. On the whole, the physical, chemical, and total exergy values of product gases are much lower than the corresponding energy values (Figure 3). This is in agreement with the findings of other researches [4, 15, 16]. The trend of exergy value is nearly the same as that of energy. The exergy values of DRS increase first and then decline (Figure 4).

### 4.4. Effect of SA/PA Ratio on Cold Gas, Energy, and Exergy Efficiencies

**Figure 5:**
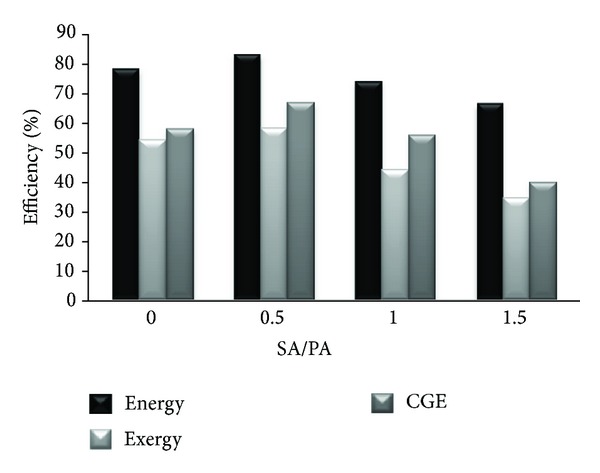
Effect of secondary to primary air ratio on cold gas, energy and exergy efficiencies.

The effect of secondary air injected to gasification zone at ER 0.195 and air flow rate of 27.93 kg/hr on cold gas, energy, and exergy efficiencies is presented in Figure 5 below. The secondary to primary air ratio is represented by (SA/PA) where SA is a secondary air flow rate measured in kg hr^−1^ injected at gasification zone and PA is a primary air flow rate in kg hr^−1^ injected below the gasifier grade.

From the same figure it is observed that a ratio of SA/PA = 0.5 is found to increase the cold gas, energy, and exergy efficiencies up 8.3%, 4.6%, and 3.6%, respectively, higher than their values at SA/PA = 0. It can be recommended that using secondary air at optimum ER for gasification process is found to increase the gasification energy and exergy efficiencies in adiabatic updraft gasifier.

## 5. Conclusions

Based on the gasification of dry refinery sludge in updraft reactor, the chemical energies of producer gases are 6.8–10.1 times higher while the chemical exergies are 2.3–5.7 times higher than the corresponding physical values. DRS has higher carbon and hydrogen content as indicated in the ultimate analysis. This therefore generates higher gaseous energy and exergy values. The cold gas, energy, and exergy efficiencies of DRS gasification are in the ranges of 22.9–55.5%, 43.7–72.4%, and 31.92–50.4%, respectively. During the process it increases initially till it reaches the maximum at the optimum ER of 0.195 and thereafter declines with further increase in ER value. The increase in ER results in more O_2_ being supplied to the gasifier, increasing oxidation process which further contributes to increase in gasification temperature. This causes a decline in the gas quality, energy, and exergy. Further, increase in the equivalence ratio injects more N_2_ with air and dilutes the producer gas, which also degrades the gas quality. When an optimum ratio of secondary air to primary air of 0.5 is injected at the gasification zone it was found that the energy values of product gases are much higher than the corresponding exergy values, which results in higher energy efficiencies.

## Figures and Tables

**Table 1:** The coefficients *a*–*d* of constant pressure, specific enthalpy and entropy at *T*_0_, and standard chemical exergy of gases.

Gas	*a*	*b* × 10^−2^	*c* × 10^−5^	*d* × 10^−9^	Range	*h*_0_ (kJ kmol^−1^)	*S*_0_ (kJ kmol^−1^ K^−1^)	ε_0,*i*_ (kJ kmol^−1^)
N_2_	28.9	−0.1571	0.8081	−2.873	273–1800	8669	191.502	720
O_2_	25.48	1.520	−0.7155	1.312	273–1800	8682	205.033	3970
H_2_	29.11	−0.1916	0.4003	−0.8704	273–1800	8468	130.574	236100
CO	28.16	0.1675	0.5327	−2.222	273–1800	8669	197.543	275100
CO_2_	22.26	5.981	−3.501	7.469	273–1800	9364	213.685	19870
CH_4_	19.89	5.024	1.269	−11.01	273–1500	—	—	831650

**Table 2:** Effect of ER on mass balance and its closure.

ER	Mass input (kg)			Mass output (kg)				Closure
	Fuel	Air	Total input	Producer gas	Char + ash	Tar	Total output	
0.167	15	18.62	33.62	32.02	0.55	0.22	32.05	0.98
0.19	15	23.27	38.27	38.65	0.47	0.19	35.70	1.02
0.195	15	27.93	42.93	41.89	0.32	0.187	39.84	0.99
0.21	15	32.58	47.58	48.98	0.35	0.194	40.26	1.04
0.22	15	37.24	52.24	45.14	0.52	0.39	35.59	0.88
0.24	15	41.89	56.89	48.17	0.265	0.479	38.46	0.86

**Table 3:** Effect of ER on energy balance and its closure.

ER	En DRS (kJ)	En air (kJ)	En gas (kJ)	En char (kJ)	En tar (kJ)	En closure
0.167	26866	781.18	9474.71	4.16	0.94	0.34
0.19	26866	889.67	15151.79	4.32	1.19	0.55
0.195	26866	911.37	20856.65	0.78	1.04	0.75
0.21	26866	1009.59	20607.39	1.19	0.80	0.74
0.22	26866	1035.78	15914.85	0.81	0.89	0.57
0.24	26866	1140.52	15933.65	0.75	0.79	0.57

## Nomenclature

En: Energy based on one kg of DRS (kJ)
Ex: Exergy based on one kg of DRS (kJ)
CGE: Cold gas efficiency
*C*_*p*_: Specific heat capacity (kJ kmol^−1^K^−1^)
*M*: Flow rate of stream k mol s^−1^
*m*_air_: Mass flow rate kg hr^−1^
*m*_*a*_: Flow rate of air m^3^hr^−1^
*m*_*D*_: Mass flow rate of DRS kg hr^−1^
*X*_ash_: Mass fraction of ash
*ε*_0,*i*_: Standard specific exergy (kJ kmol^−1^)
*a*, *d*: Coefficients of constant pressure specific heat capacity
*T*: Temperature (K)
*T*: Duration time (hr or min)
*P*: Pressure (Pa)
*Z*: Height (m)
*G*: Gravitational acceleration (ms^−2^)
*S*: Specific entropy (kJ kmol^−1^K^−1^)
*H*: Specific enthalpy (kJ kmol^−1^)
*C*, *S*: Weight fractions in ultimate analysis
*Y*: Fuel gas production (N m^3^ kg^−1^)

### Abbreviations

HHV: High heating value (MJ kg^−1^)
LHV: Low heating value (MJ kg^−1^)
ER: Equivalent ratio
AF: Air fuel ratio

### Creek Letters

*Β*: Correlation factor
Δ*H*: Change in the system enthalpy kJ kg^−1^
*H*: Efficiency

### Superscripts

Ch: Chemical
Ph: Physical
To: Total
Po: Potential
0: Standard
Ki: Kinetics

### Subscripts

0: Ambient condition
Air: Related to air
Uc: Related to unreacted carbon
Loss: Related to the lost
Tar: Related to tar
Gas: Related to gases
DRS: Related to dry refinery sludge
Ash: Related to ash
Stoich: Stoichiometric conditions.
